# Dental Colleges

**Published:** 1863-10

**Authors:** 


### Editorial.

### DENTAL COLLEGES.

BALTIMORE DENTAL COLLEGE.—This, the oldest Dental College in the world, has made its annual announcement for its twenty-third session. It has a reputation and a favorable one too, throughout the world where dental science is known. It has well sustained itself through all the vicissitudes that have come upon it, and these have been neither few nor small; we refer to such things as necessarily come upon all similar institutions, and such as all the others have experienced in proportion to their age. We have had much solicitude for this Institution during the last three years, being situated in a portion of the country where peril and trouble were constantly imminent. There was danger that it would be lost amid the whirl of wild confusion that seemed to reign almost supreme. But we are glad that it survives it all, and now it *will* live. It has done so, however, only through the energy and perseverance of those having it in charge. We know that those who place themselves under its fostering care, will not have occasion to regret it.

OHIO COLLEGE OF DENTAL SURGERY.—This, the second Dental School in the country, and indeed in the world, is about to open its eighteenth session. It, like others, has been subject to changes, through which it has passed. It has kept up its regular courses, from the beginning to the present, with one interruption. The results of its operations and influence is perhaps exhibited, in at least, not an unfavorable aspect, by a large proportion of those who have gone out as its alumni.

This institution, in a pecuniary point of view, is in no respect inferior to its compeers. It has property to a considerable amount, appropriated to its own sustenance. The building and ground are to be used for the purposes of the College, so long as the institution shall exist: this adds greatly to its permanency. We should be glad to see every other dental college established upon a similar basis.

PENNSYLVANIA COLLEGE OF DENTAL SURGERY.—We received the announcement of this Institution some time ago, in connection with the "Dental Times," and we can not do less than express our gratification, that so good an organization has been effected. We enjoyed the privilege, a short time ago, of visiting this Institution, and examining its whole arrangement; it is well adapted in all respects for the accomplishment of the work to be done. Every department is well supplied with appropriate apparatus.

This Institution has also had its changes, through which it has passed, seeming to retain its prestige, power and popularity.

PHILADELPHIA DENTAL COLLEGE.—This is a new Institution. We have recieved the announcement for the first session, and we feel well assured that the organization is such as will prove eminently effective. The feasability of two dental colleges in Philadelphia, has been questioned; but that they will both succeed, if well managed, we have no doubt. Philadelphia is one of the most favorable locations for medical Institutions in the country. It has been suggested that there are already more dental colleges than have been well sustained, and that increasing the number will only augment the difficulty.

We think it will be found that the former schools will be quite as well, if not better sustained than before, and that the new Institution will develope its own material and work. All will admit that there is work enough for four, or even more institutions of this kind; and it has not been done, because attention has not been excited and directed to it. There are hundreds, perhaps thousands, in the country, who have been attempting to practice—have made a small beginning,—who ought to take a course in a dental college, and many of them would if they were *waked up*. The addition of one or two more schools would aid materially in this work; and entertaining this view, we hail the new school as an instrumentality of good to the profession: it will not materially affect those already in operation, except perhaps to stimulate them to greater energy.

We would venture the suggestion, that at least three additional schools are demanded by the best interests of the profession; we would locate them, one in New York, one in Boston, and another at some point in the West. New England *alone*, ought to sustain two colleges, and New York and its vicinity, another. T.

